# Effects of a Proprioceptive Training Program on Dynamic Balance and Neuromotor Performance in Adolescent Latin American Dancers

**DOI:** 10.3390/sports13110388

**Published:** 2025-11-04

**Authors:** Nicola Mancini, Siria Mancini, Miriana Ferrantino, Fiorenzo Moscatelli, Giovanni Messina, Marcellino Monda, Maria Ruberto, Paride Vasco, Claudia Casella, Francesco Paolo Colecchia, Antonietta Messina, Rita Polito

**Affiliations:** 1 Department of Education and Sport Sciences, Pegaso Telematic University, 80143 Naples, Italy; nicola.mancini@unipegaso.it (N.M.); maria.ruberto@unipegaso.it (M.R.); 2 Department of Clinical and Experimental Medicine, University of Foggia, 71121 Foggia, Italy; siria_mancini.585707@unifg.it (S.M.); ferrantino.miriana@gmail.com (M.F.); 3 Department of Experimental Medicine—Section of Human Physiology and Unit of Dietetics and Sports Medicine, University of Campania “Luigi Vanvitelli”, 80138 Naples, Italy; giovanni.messina@unicampania.it (G.M.); marcellino.monda@unicampania.it (M.M.); francescocolecchia@gmail.com (F.P.C.); 4 Department of Humanistic Studies, University of Foggia, 71121 Foggia, Italy; paride.vasco@unifg.it; 5 Department of Advanced Biomedical Sciences—Section of Legal Medicine, University of Naples Federico II, 80138 Naples, Italy; claudia.casella@unina.it; 6 Department of Precision Medicine, University of Campania “Luigi Vanvitelli”, 80138 Naples, Italy; antonietta.messina@unicampania.it; 7 Department of Psychology and Health Sciences, Pegaso Telematic University, 80143 Naples, Italy; rita.polito@unipegaso.it

**Keywords:** dynamic balance, Latin American dance, adolescent dancers, proprioceptive training, Y Balance Test, Pediatric Reach Test, Single-Leg Landing Stability Test, countermovement jump, postural control, functional symmetry

## Abstract

Balance is a key determinant of movement quality and injury prevention in dance, yet targeted dynamic-balance training is rarely embedded in adolescent curricula. This controlled experimental study evaluated a 10-week proprioceptive add-on protocol integrated into Latin American dance practice on neuromotor performance in adolescent female dancers. One hundred twenty-four participants were allocated to an experimental group (EG; *n* = 62) or a control group (CG; *n* = 62). Outcomes were the Y Balance Test (YBT; composite and inter-limb asymmetry), Pediatric Reach Test (PRT; anterior and lateral), Single-Leg Landing Stability Test (SLLST; time to stabilization), and countermovement jump (CMJ; bilateral and single-leg). The EG completed 25–30 min of progressive balance work twice weekly before class, while the CG continued standard technical training with matched volume. Compared with the CG, the EG showed clear pre–post improvements in YBT (bilateral composite increased; asymmetry decreased), PRT (anterior and lateral increased), CMJ (bilateral and right single-leg increased), and SLLST (time to stabilization decreased), with significant group × time interactions across domains. Specifically, improvements were significant for Y Balance Test composite scores (*p* < 0.001), Pediatric Reach Test (*p* ≤ 0.01), countermovement jump (*p* < 0.05), and time to stabilization (*p* ≤ 0.01), confirming robust within- and between-group effects. These findings indicate specific neuromotor adaptations attributable to the integrated protocol. Beyond performance enhancement, the proprioceptive program may contribute to injury prevention, better postural efficiency, and safer execution of complex dance movements in adolescent dancers.

## 1. Introduction

Balance results from a complex integration of sensory, neuromuscular, and cognitive systems and is a prerequisite for movement quality and injury prevention in performance settings such as dance [1,2,3,4]. In dancers, postural control is refined through the combined processing of visual, vestibular, and somatosensory information, with discipline-specific evidence of adaptive strategies in the visual [5,6], vestibular [7], and proprioceptive domains [8]. During adolescence, sensorimotor organization undergoes substantial refinement, involving re-weighting between visual, vestibular, and proprioceptive inputs that affects balance and coordination. In dancers aged approximately 13–15 years, these adaptations are further shaped by continuous motor practice and postural specialization.

Latin American dances (e.g., Cha-cha, Samba, Rumba, Paso Doble, Jive) are characterized by high tempo, rapid changes in direction, rotations, weight transfers, and partner work—elements that impose high demands on dynamic balance, core control, and functional symmetry [9,10,11]. Recent findings show that experienced Latin dancers display greater gait symmetry and better single-leg stability than physically active non-dancers, supporting a potential training effect of the discipline on coordination and balance [12,13,14], as well as general training benefits in related student populations [15].

Moreover, structured neuromuscular/proprioceptive programs are effective in improving stability and postural-control indicators in dancer populations [16,17,18], in line with the well-documented relationship between balance and athletic performance [19] and with established balance-training principles [20].

Despite this context, experimental studies investigating the impact of a dance-specific training protocol on dynamic balance in adolescent female Latin dancers remain scarce, particularly those employing controlled designs, pre–post-measurements, and validated tools (e.g., the Y Balance Test, including the asymmetry index) alongside landing/re-stabilization tasks [21,22]. This gap has practical implications: in schools and clubs, technical programs are often centered on choreography and discipline-specific skills, whereas systematic, differentiated routines for dynamic-balance training are lacking—limiting an evidence-based pathway for young dancers [9,20].

### Study Aim

Building on these premises, the present study evaluates the effectiveness of a training protocol targeting dynamic balance, integrated into Latin American dance practice, in young female dancers. Specifically, we examine whether the intervention yields measurable improvements in (i) Y Balance Test performance (including percentage asymmetry of the composite score), (ii) time-to-stabilization during functional single-leg landings (SLLST), and (iii) stretch-shortening explosive strength (single- and bilateral countermovement jumps), compared with a control group. The resulting evidence is intended to inform teaching and coaching practice by providing operational guidance for integrating dynamic-balance training into adolescent dancers’ curricula [17,20,23,24]. During adolescence, Latin American dancers experience rapid physical and neuromotor changes that can transiently affect coordination and postural control. Growth-related alterations in limb length, muscle strength, and motor timing may increase asymmetries and the risk of overuse injuries, especially under repetitive, high-impact conditions typical of Latin styles. Moreover, the technical demands of these dances—requiring precise weight transfers, rapid turns, and sustained single-leg supports—make targeted balance training particularly relevant to ensure safe motor development and injury prevention [25,26,27]. From a practical and health-oriented perspective, suboptimal balance and insufficient postural control may lead to decreased movement precision, greater joint loading, and increased vulnerability to lower-limb injuries. Over time, these factors can compromise both technical performance and the long-term development of efficient, symmetrical motor patterns, highlighting the need for structured proprioceptive interventions during this critical developmental stage.

## 2. Materials and Methods

### 2.1. Study Design

This controlled experimental study employed two parallel groups—an experimental group (EG) and a control group (CG)—with pre–post-assessments over ten weeks (T0, pre-test; T1, post-test). The aim was to evaluate the effectiveness of a training protocol specifically targeting dynamic balance, integrated into Latin American dance practice, on the neuromotor capacities of adolescent female dancers.

We compared T0 and T1 in EG and CG for the following outcomes: Y Balance Test (YBT) composite score on both limbs (and percentage inter-limb asymmetry), Pediatric Reach Test (PRT) reach distance (anterior and lateral), Single-Leg Landing Stability Test (SLLST) time to stabilization, and countermovement jump (CMJ) height performed bilaterally and unilaterally.

### 2.2. Participants and Eligibility Criteria

A total of 124 adolescent female Latin dancers were enrolled and evenly assigned to the experimental group (EG; *n* = 62) or the control group (CG; *n* = 62). Assessments were conducted at baseline (T0) and after 10 weeks (T1). Mean age at T0 was 14.5 ± 0.6 years in EG and 14.7 ± 0.7 years in CG (inclusion range: 13–15 years). Baseline anthropometrics and age are reported in Table 1. Participants were recruited via non-probability convenience sampling from Latin American dance schools in Southern Italy that guaranteed regular attendance; all participants completed both assessment sessions. Participants were allocated to groups using a quasi-random procedure based on enrollment order (alternate assignment to Experimental or Control Group), ensuring balanced sample sizes (*n* = 62 per group) and comparable baseline characteristics. An a priori power analysis conducted with G*Power 3.1 indicated that a total sample of at least 98 participants (49 per group) would be required to detect a medium effect size (f = 0.25) for the group × time interaction in repeated-measures ANOVA with α = 0.05 and power (1 − β) = 0.80. The achieved sample of 124 participants thus provided adequate statistical power.

#### 2.2.1. Inclusion Criteria

- ≥2 years of continuous participation in the discipline;
- absence of neurological or vestibular disorders;
- absence of acute musculoskeletal conditions;
- attendance ≥80% of scheduled sessions;
- written informed consent from legal guardians.

#### 2.2.2. Exclusion Criteria

- missing data at T0 or T1;
- body mass index (BMI) < 15 or >25 kg/m^2^;
- 20% absences during the experimental period.

### 2.3. Training Protocol

Baseline (T0) and post-intervention (T1) assessments were conducted outside regular class hours at the same facility and were preceded by a standardized 10 min warm-up. In addition to their usual technical training, the experimental group (EG) performed a balance-focused add-on program lasting 25–30 min, twice per week, immediately before the dance class, for ten consecutive weeks (20 total sessions). Load parameters, including exercise duration, repetitions, rest intervals, and weekly progression, are comprehensively detailed in Table 2 and Table 3 to ensure transparency and reproducibility of the conditioning stimulus. The protocol was designed in accordance with established proprioceptive and neuromuscular training approaches validated in youth and dancer populations [21,22,23,28,29], ensuring scientific consistency and reproducibility.

The program comprised:

- Unstable-surface work: balance/wobble boards, foam/air cushions, and elastic or mini-trampoline mats;
- Dynamic stability tasks: balance walking, rotations, changes in base of support;
- Single-leg drills (static and dynamic): controlled leg swings, single-leg squats, lunge-to-jump transitions;
- Postural control and core stability: e.g., the “airplane” exercise (single-leg trunk/hip extension with controlled alignment);
- Rapid transitions with reaching tasks;
- Contextual variability: partner-based drills and station circuits, when appropriate, to enhance task adaptability.

Difficulty was progressively increased by introducing higher levels of instability, controlled external perturbations, and dual- and multi-task demands with visual or cognitive stimuli. Load structure and detailed drill progressions are detailed in Table 2 and Table 3.

The control group (CG) continued their standard technical program with no specific balance add-on; total weekly training volume (hours) was matched between groups.

Detailed drill lists for EG are provided in Table 3.

**Table 3:** Weekly proprioceptive/balance protocol for the Experimental Group (EG): static and dynamic drills and progressions.

Week(s)	EG—Balance/Proprioceptive Add-on (25–30 min Before Class, 2×/Week)
1–2	Foundations on unstable support: bi-/uni-pedal stance on foam/air cushions (eyes open/closed); ball pick-ups from floor while maintaining balance; partner elastic-band resistance on cushion. Simple locomotor balance: single-leg hops with return on the opposite leg; lateral hops over a rope; “airplane” (single-leg trunk/hip extension). alternate stance leg; small controlled hops → faster tempo; rhythm changes; eyes-closed variants.
3–4	Directional hopping: jumps along 5 spaced pads (≈1 m) alternating single-leg landings; same in zig-zag. Dual-task balance: keep a balloon aloft while standing on cushion. Landing control: trampoline/elastic-mat jumps with stick landing. Support transitions: “airplane” on blocks; forward block-exchange stepping drill. *Progressions*: increase speed/amplitude; eyes-closed trials; increase consecutive jumps/hold time.
5–6	Single-leg stability on cushion: eyes open/closed; partner ball passes (bi-/uni-pedal); “airplane” on cushion. Cooperative balance: paired circle hops holding one partner’s leg; balance “duels” on blocks. Intro plyometrics: squat jumps ×5; lunge-to-jump ×5. *Progressions*: add free-leg movements; perform passes in single-leg stance; shorten recovery; increase reps/series; add controlled perturbations.
7–8	Refinement (weeks 1–2 repeated at higher precision): single-leg on cushion eyes open/eyes closed; ball pick-ups (bi-/uni-pedal); partner elastic resistance. Rotational control: forward/return rope hops; single-leg rotations with stop in balance; “airplane” with forefoot stepping. *Progressions*: longer holds; stronger elastic traction; faster hops; eyes-closed arrests.
9–10	Integration and complexity phase. Linear and zig-zag pad jumps with ball dribble/taps; “airplane” on blocks with eyes closed plus tactile/visual cues; paired balance challenges with tactile prompts (eyes closed); coordinated ball passes on unstable surfaces with circular free-leg motions; plyometric tasks with reduced visual input. *Progressions*: shorter rests, higher speed, varied arm positions; maximal neuromotor control under destabilization.

Note. Add-on performed twice weekly, 25–30 min before the technical class (see Table 4). Drills are typically organized in 2–3 mini-circuits, each 2–3 sets of 20–40 s per set (or 8–12 repetitions) with 20–40 s recovery, scaled to group logistics. Equipment: foam/air cushions, elastic bands, small blocks/bricks, rope or chalk line, soft ball/balloon, trampoline/elastic mat. Safety and execution cues: progressive exposure to eyes-closed; supervised partner tasks; controlled perturbations; neutral trunk/pelvis alignment; when relevant, hands on iliac crests to limit arm compensation. Control group (CG) continued the unchanged technical/traditional program with matched weekly volume (see Table 2).

**Table 4:** Descriptive statistics (mean ± SD) at baseline (T0) and post-intervention (T1) for the Experimental Group (EG) and Control Group (CG).

Variable	EG T0	EG T1	CG T0	CG T1
Composite Score Right (%)	79.26 ± 6.72	94.72 ± 8.07	76.85 ± 9.00	77.09 ± 7.99
Composite Score Left (%)	80.93 ± 7.42	94.25 ± 9.53	77.26 ± 9.23	78.76 ± 8.59
Asymmetry Composite (%)	−1.68 ± 2.10	0.47 ± 2.37	−0.41 ± 4.89	−1.68 ± 3.35
PRT_Anterior (cm)	25.09 ± 4.57	29.87 ± 4.27	24.19 ± 2.68	23.39 ± 2.75
PRT_Lateral Right (cm)	19.85 ± 5.65	24.60 ± 4.91	17.57 ± 3.23	16.19 ± 3.35
PRT_Lateral Left (cm)	20.89 ± 4.17	25.08 ± 4.12	18.06 ± 4.42	17.41 ± 2.92
CMJ (cm)	19.67 ± 4.01	21.30 ± 4.41	20.14 ± 2.45	19.82 ± 2.70
CMJ_Single Right (cm)	8.17 ± 1.95	10.05 ± 1.96	9.62 ± 2.63	9.48 ± 2.23
CMJ_Single Left (cm)	9.59 ± 2.54	9.92 ± 2.31	8.51 ± 0.82	7.66 ± 2.04
SLLST_Right (s)	5.08 ± 1.84	3.84 ± 1.44	5.15 ± 1.86	5.22 ± 2.65
SLLST_Left (s)	5.15 ± 1.87	4.00 ± 1.92	5.30 ± 1.80	5.57 ± 1.73

Note. Data are mean ± SD. YBT (Composite and Asymmetry), PRT (Anterior, Lateral Right, Lateral Left), CMJ (bilateral and single-leg), and SLLST (right and left) outcomes are reported. Units: YBT Composite and Asymmetry are reported as percent of leg-length-normalized reach (ASIS–medial malleolus); PRT distances in centimeters (cm); CMJ heights in centimeters (cm); SLLST stabilization time in seconds (s). Lower SLLST values indicate better performance. Abbreviations: YBT = Y Balance Test; PRT = Pediatric Reach Test; CMJ = Countermovement Jump; SLLST = Single-Leg Landing Stability Test; EG = Experimental Group; CG = Control Group.

### 2.4. Ethics Statement

All participants and their legal guardians provided written informed consent. The study was conducted in accordance with the Declaration of Helsinki and was approved by the Ethics Committee of Università Telematica Pegaso (protocol code PROT/E 002466; approval date: 29 March 2024).

### 2.5. Outcome Measures

#### 2.5.1. Y Balance Test (YBT)

The Y Balance Test (YBT) was used as a measure of dynamic balance. It is the instrumented version of components of the Star Excursion Balance Test (SEBT), validated by Plisky et al. [28,29], with high reliability (ICC > 0.85) and good predictive validity in youth and athletic populations. Testing was performed with the Y-Balance Test (Y-BT) kit, consisting of a central platform and three graduated sliding reach indicators oriented in the anterior (ANT), posteromedial (PM), and posterolateral (PL) directions.

*Procedure.* Dancers were tested barefoot, with hands placed on the iliac crests, standing on one leg on the central platform. The stance foot was aligned with the “zero” mark of the active reach arm. With the non-stance limb, each participant gently pushed the slider along the required direction, then returned under control to the initial upright position. A fixed order of directions was adopted (anterior → posteromedial → posterolateral) and, for each direction, trials were performed in the sequence right limb → left limb. Three valid attempts were recorded for each limb and direction, with brief rest between attempts to avoid fatigue. For each direction, the best valid value across the three attempts was used.

*Invalid trial criteria.* A trial was discarded and repeated if the participant: (i) lost single-leg stance or touched the floor with the reaching foot; (ii) kicked the slider (i.e., no controlled contact) or used it for support; (iii) failed to keep the hands on the iliac crests; or (iv) did not return the reaching foot to the starting position under control. Distances were read at the edge of the indicator at the point of maximal controlled excursion.

*Normalization.* Distances were normalized to lower-limb length (LL), measured in the supine position from the anterior superior iliac spine (ASIS) to the medial malleolus; if two measurements differed by >0.5 cm, a third measurement was taken and the mean used. For each direction:

$$Normalized\;Reach\;direction\left(\%\right)=\frac{Reach\;Distancedirection}{LL}\times 100$$

Composite score (per limb, %)

$$Composite\left(\%\right)=\frac{ANT+PM+PL}{3\times LL}\times 100$$

where ANT, PM, and PL are the best valid reach distances (in cm) in the three directions, and LL is expressed in cm.

Functional asymmetry (signed)

$$Asymmetry\left(\%\right)=Composite\;Right-Composite\;Left$$

Positive values indicate superior right-limb performance; negative values indicate superior left-limb performance. For analysis, we used the right/left composite scores (%) and the asymmetry (%). Procedures and test orders were identical at T0 and T1 to minimize learning effects [29].

#### 2.5.2. Pediatric Reach Test (PRT)

The standing Pediatric Reach Test (PRT) was used to assess anticipatory postural control. Adapted from the Functional Reach Test and validated in pediatric populations [30], it measures the maximal voluntary excursion forward (and laterally, when applied) with the dominant arm extended at 90° of shoulder flexion, without loss of balance. Each condition (anterior, right lateral, left lateral) was performed with three attempts, and the best valid value was recorded. Trials lacking control (e.g., loss of alignment, taking an additional step) were discarded and repeated. The test has been shown to be a reliable and age-appropriate measure of anticipatory postural control in children and adolescents [30].

#### 2.5.3. Single-Leg Landing Stability Test (SLLST)

Post-landing stability was assessed using the Single-Leg Landing Stability Test (SLLST). In accordance with prior experimental applications [31], a triaxial accelerometer (FreeSense^®^, Sensorize, s.r.l., Rome; Italy; 200 Hz) was positioned at the L4 level using an elastic belt. The task consisted of a forward jump from a line set at 50% of body height, landing on the same limb and maintaining the position for at least 5 s. Time to stabilization (TTS) was computed as the post-impact interval during which the variance of the vector magnitude of triaxial acceleration remained below 0.005 g^2^ for at least 0.5 s consecutively, in line with Wiese et al. [32], Fransz et al. [33], and Liu et al. [13].

#### 2.5.4. Countermovement Jump (CMJ)

Explosive stretch–shortening performance was assessed using the countermovement jump (CMJ) on a Chronojump-Boscosystem^®^, ver. 2.5.2-63, contact (conductive) jump mat with dedicated software, following the C. Bosco protocol. The CMJ shows strong validity and reliability in physically active populations [34,35], and Chronojump measurements demonstrate high test–retest and inter-device reliability [36].

Procedure: Each dancer performed three bilateral and three single-leg CMJs (right and left tested separately), hands on hips with no arm swing, and a self-selected countermovement depth. Invalid attempts (e.g., tucking in flight, uncontrolled landings) were discarded and repeated. Jump height was derived from flight time using the classical equation: h = (g × t^2^)/8 (where h = jump height, g = 9.81 m·s^−2^, and t = flight time), assuming comparable posture at take-off and landing [37]. For analysis, the best valid trial was retained. The CMJ has strong validity and reliability in physically active populations [35], and Chronojump measurements show excellent test–retest and inter-device reliability [36].

### 2.6. Data Collection Procedures

All assessments were conducted in familiar dance studios under controlled ambient temperature and at times compatible with the participants’ training routines. All motor tests were performed barefoot to standardize execution and eliminate any potential variability related to footwear or heel height. Before baseline testing (T0), all participants completed a supervised familiarization session covering the standardized warm-up and the main testing procedures (YBT, PRT, SLLST, and CMJ). Each dancer practiced the required movements until demonstrating stable and safe execution. This preliminary session aimed to reduce learning effects and ensure consistent performance quality across participants.

At each session (T0; T1), body height and mass were measured using a portable stadiometer seca 213 (seca GmbH & Co. KG, Hamburg, Germany; resolution 0.1 cm) and a flat digital scale seca 874 (seca GmbH & Co. KG, Hamburg, Germany; resolution 0.1 kg). Anthropometrics were taken barefoot, in light clothing, and without metallic objects; each measurement was taken twice (a third time if the difference exceeded 0.5 cm for height or 0.2 kg for mass), and the mean was recorded. Subsequently, tests were administered in a fixed order—YBT → PRT → SLLST → CMJ—to minimize learning or fatigue effects, maintaining standardized execution conditions across T0 and T1. This specific sequence was adopted to minimize cumulative fatigue and ensure postural stability before executing more demanding tasks, in accordance with previous experimental protocols in youth and dancer populations [29,31]. Counterbalancing was avoided because randomizing tasks of different physical intensities (e.g., landing vs. jumping) could have introduced unwanted fatigue or motor interference effects. The reliability of the three trials for each test was high in the present protocol (intraclass correlation coefficients, ICC = 0.87–0.95 across variables), consistent with published test–retest values reported for YBT, PRT, SLLST, and CMJ [28,29,30,31,32,33,34,35,36]. All evaluations were performed by trained assessors (degree in Sport and Exercise Sciences) following standard operating procedures (SOPs) and standardized instructions, under the on-site supervision of the lead investigator.

### 2.7. Statistical Analysis

Analyses were performed in SPSS v.25.0 (IBM, Chicago, IL, USA). Preliminary checks included detection of outliers, assessment of the normality of difference scores (Δ = T0–T1) via the Shapiro–Wilk test and Q–Q plot inspection, and evaluation of homogeneity of variances using Levene’s test for between-group comparisons. Under the Δ = T0–T1 convention, improvements that increase the raw metric (YBT, PRT, CMJ) yield negative t and d_z_ values, whereas improvements that decrease the metric (SLLST) yield positive t and d_z_. For each variable, we computed the mean, standard deviation, 95% confidence interval (95% CI), and descriptive Δ (T0–T1). Within-group changes (T0 vs. T1) were tested with paired *t*-tests, reporting effect sizes as Cohen’s d_z_ = t/√n (with *n* = 62). Between-group comparisons (EG vs. CG) at T1 were conducted with independent-samples *t*-tests when homoscedasticity was met. The specific effect of the intervention was evaluated using a 2 × 2 repeated-measures ANOVA (within-factor: time; between-factor: group); the group × time interaction is reported as F(1,122) with partial eta-squared *η_P_*^2^ as the effect-size index. Statistical significance was set at *p* < 0.05. All analyses were verified through sensitivity checks (e.g., Wilcoxon signed-rank tests and Holm–Bonferroni adjustment), detailed in the Results section.

## 3. Results

### 3.1. Sample and Adherence

All 124 enrolled dancers completed the protocol (EG = 62; CG = 62), with no adverse events or drop-outs. Baseline characteristics were comparable between groups (all *p* > 0.05; Table 1).

### 3.2. Variable Trajectories and Within-Group Comparisons

Descriptive T0–T1 data (mean ± SD) are reported in Table 4. In the EG, we observed substantial improvements in dynamic balance (YBT), anticipatory postural control (PRT), explosive strength (CMJ), and post-landing stability (SLLST), whereas changes in the CG were minimal or absent. In paired tests (Δ = T0–T1), the EG showed significant increases in the YBT composite score for the right limb (*t* = −10.214, *p* < 0.001, d_z_ ≈ 1.30) and left limb (*t* = −6.847, *p* < 0.001, d_z_ ≈ 0.87), along with a reduction in asymmetry (*t* = −2.350, *p* = 0.038, d_z_ ≈ 0.30). PRT performance improved in the anterior direction (*p* = 0.002) and in right/left lateral reach (*p* = 0.006/0.009).

For explosive strength, increases were observed in the single-leg CMJ on the right (*t* = −4.883, *p* < 0.001, d_z_ ≈ 0.62) and in the bilateral CMJ (*t* = −2.327, *p* = 0.040, d_z_ ≈ 0.30), whereas the single-leg CMJ on the left did not reach significance. In post-landing stabilization tasks (SLLST), times decreased for the right (*t* = 3.711, *p* = 0.003, d_z_ ≈ 0.47) and left limb (*t* = 3.246, *p* = 0.008, d_z_ ≈ 0.41), indicating improved postural control. No significant changes were detected in the CG.

**Figure 1:**
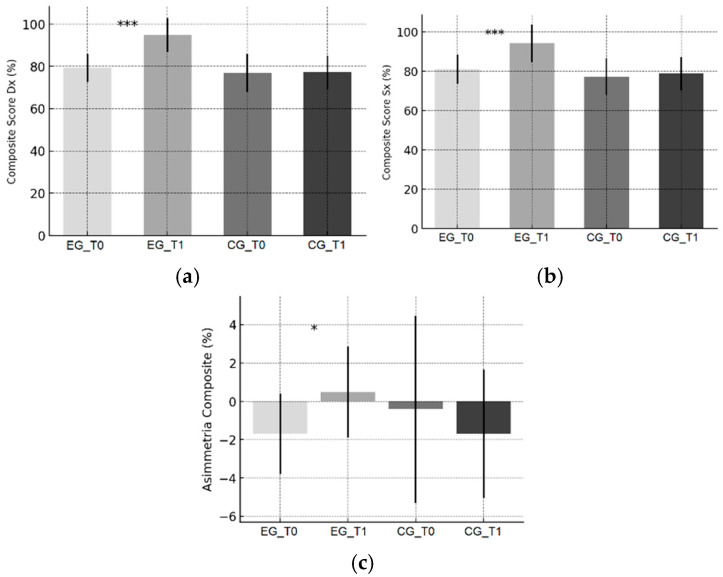
Y Balance Test outcomes. (**a**) Composite score—right limb; (**b**) Composite score—left limb; (**c**) Composite-score asymmetry (Right–Left, %). Bars show mean ± SD at baseline (T0) and post-intervention (T1) for the Experimental Group (EG) and Control Group (CG). Distances are normalized to leg length (ASIS–medial malleolus). Significance of within-group T0–T1 changes (paired *t*-tests): * *p* < 0.05, *** *p* < 0.001. In panel (**c**), values closer to 0 indicate greater inter-limb symmetry; positive values favor the right limb.

**Figure 2:**
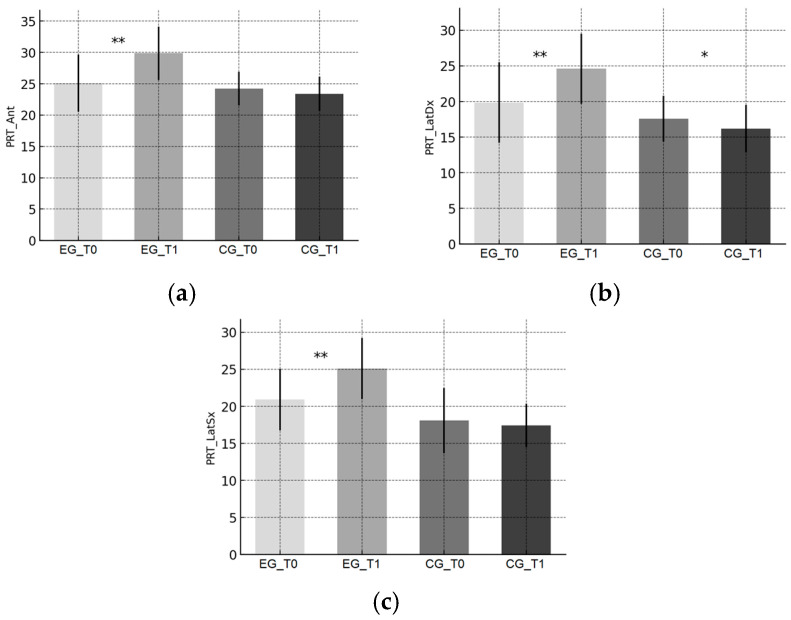
Pediatric Reach Test (PRT) outcomes. (**a**) Anterior reach; (**b**) Lateral reach—right; (**c**) Lateral reach—left. Bars show mean ± SD at baseline (T0) and post-intervention (T1) for the Experimental Group (EG) and Control Group (CG). Distances are reported in centimeters. Significance of within-group T0–T1 changes (paired t-tests): * *p* < 0.05, ** *p* < 0.01.

**Figure 3:**
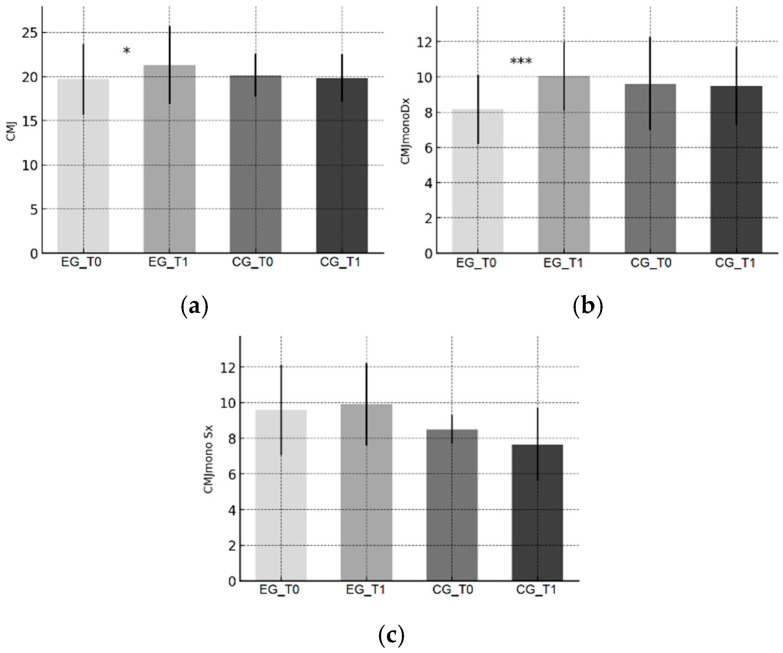
Countermovement Jump (CMJ) performance. (**a**) Bilateral CMJ height; (**b**) Single-leg CMJ—right; (**c**) Single-leg CMJ—left. Bars show mean ± SD at baseline (T0) and post-intervention (T1) for the Experimental Group (EG) and Control Group (CG). Heights are reported in centimeters. Significance of within-group T0–T1 changes (paired *t*-tests): * *p* < 0.05, *** *p* < 0.001.

**Figure 4:**
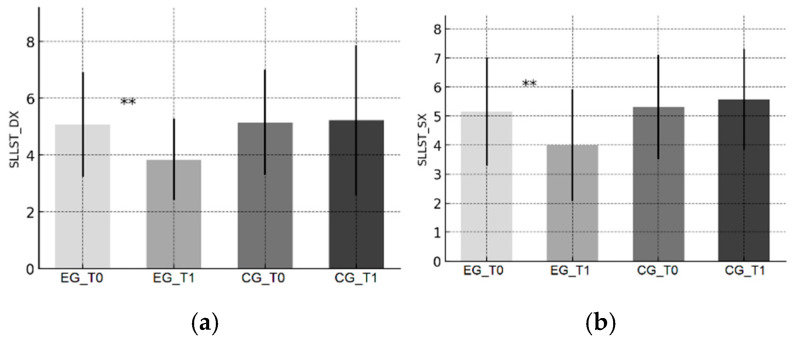
Single-Leg Landing Stability Test (SLLST)—stabilization time. (**a**) Right limb; (**b**) Left limb. Bars show mean ± SD at baseline (T0) and post-intervention (T1) for the Experimental Group (EG) and Control Group (CG). Times are reported in seconds; lower values indicate better performance. Significance of within-group T0–T1 changes (paired *t*-tests): ** *p* < 0.01.

Trends are illustrated in Figure 1a–c (YBT), Figure 2a–c (PRT), Figure 3a–c (CMJ), and Figure 4a,b (SLLST).

### 3.3. Group × Time Interaction (Two-Way Repeated-Measures ANOVA)

The repeated-measures ANOVA revealed significant group × time interactions in favor of the EG for:

YBT composite—right: F(1,122) = 43.534, *p* < 0.001, *η_P_*^2^ = 0.263; left: F(1,122) = 25.929, *p* < 0.001, *η_P_*^2^ = 0.175;
PRT—anterior: F(1,122) = 19.284, *p* < 0.001, *η_P_*^2^ = 0.136; right lateral: F(1,122) = 16.357, *p* < 0.001, *η_P_*^2^ = 0.118; left lateral: F(1,122) = 10.327, *p* = 0.004, *η_P_*^2^ = 0.078;
CMJ—single-leg right: F(1,122) = 7.676, *p* = 0.011, *η_P_*^2^ = 0.059; bilateral: F(1,122) = 5.307, *p* = 0.031, *η_P_*^2^ = 0.042;
SLLST—right: F(1,122) = 6.190, *p* = 0.021, *η_P_*^2^ = 0.048; left: F(1,122) = 5.315, *p* = 0.031, *η_P_*^2^ = 0.042;
YBT asymmetry: F(1,122) = 4.858, *p* = 0.038, *η_P_*^2^ = 0.038.

Details are reported in Table 5.

### 3.4. Summary Relative to the Hypotheses

The hypotheses were supported: in the EG, dynamic balance (YBT) and functional symmetry improved, explosive strength increased (bilateral CMJ and right single-leg CMJ), and time to stabilization (SLLST) decreased. The left single-leg CMJ showed a favorable but non-significant trend. Paired-test statistics are reported in Table 6; ANOVA interactions are shown in Table 5.

#### Sensitivity Analyses

Assumptions of normality were assessed using the Shapiro–Wilk test and visual inspection of Q–Q plots. Because the paired *t*-test requires normality of the difference scores (Δ = T0–T1), Δ was examined for each variable. In the presence of minor deviations, results were confirmed with the Wilcoxon signed-rank test, which preserved the direction and significance of the main findings. Given multiple comparisons, the primary outcomes (YBT composite/asymmetry, SLLST, CMJ, PRT) were pre-specified; as an additional safeguard, a Holm–Bonferroni post hoc correction did not alter the substantive conclusions.

### 3.5. Interpretation of Results—Hypothesis Testing

- H1. In the EG, YBT composite scores increased markedly for both limbs (d_z_ ≈ 1.30 and 0.87), with significant group × time interactions [F(1,122) = 43.534 and 25.929; *η_P_*^2^ = 0.263 and 0.175].
- H2. YBT asymmetry decreased significantly [F(1,122) = 4.858; *η_P_*^2^ = 0.038].
- H3. Explosive strength improved in the bilateral CMJ and the right single-leg CMJ (small–moderate effects); the left single-leg CMJ showed only a non-significant favorable trend.
- H4. Time to stabilization (SLLST) decreased significantly on both sides [F(1,122) = 6.190 and 5.315; *η_P_*^2^ = 0.048–0.042].

PRT (anterior and lateral) also improved with robust interactions (*η_P_*^2^ ≈ 0.08–0.14), indicating enhanced anticipatory postural control.

Overall, the intervention elicited specific neuromotor adaptations in dynamic balance, functional symmetry, and post-landing stabilization, with clear implications for training design in adolescent dancers. Statistical details are provided in Table 7.

## 4. Discussion

### 4.1. Confirmation or Refutation of the Hypotheses

The primary aim of this study was to examine the effects of a proprioceptive training protocol, integrated into Latin American dance practice, on dynamic balance, anticipatory postural control, and related functional indices (post-landing stabilization, SLLST; explosive strength, CMJ) in adolescent female dancers. In the Experimental Group (EG), significant improvements emerged in the Y Balance Test (YBT) composite score, in all three directions of the Pediatric Reach Test (PRT), in jump height (bilateral CMJ and right single-leg CMJ), and in time to stabilization (SLLST), together with a reduction in YBT functional asymmetry. The Control Group (CG) maintained essentially stable performance. The group × time interactions in the 2 × 2 ANOVA confirm that these improvements are attributable to the intervention (see Table 6), indicating specific neuromotor adaptations even in already trained participants.

### 4.2. Comparison with the Literature and Implications

Here we interpret the findings in light of prior literature and discuss implications for competitive dance. The analysis is organized by the functional domains assessed: dynamic balance and symmetry (YBT), anticipatory postural control (PRT), stretch–shortening explosive strength (CMJ), and post-landing stabilization (SLLST). For each outcome, we relate effect magnitudes, consistency with previous studies, and implications for performance and injury prevention. Given the specific biomechanical demands of Latin American dance (rapid turns, weight transfers, single-leg supports) and the adolescent age range of our sample (13–15 years), proprioceptive training likely exploited a developmental window of high neuromotor adaptability, amplifying the effectiveness of the intervention.

#### 4.2.1. Y Balance Test (Dynamic Balance and Functional Symmetry)

In the EG, the YBT composite score increased markedly for both limbs, with a clear reduction in functional asymmetry (Figure 1a–c). These outcomes are consistent with the high reliability and sensitivity of the YBT reported in the literature [28,29,38] and with previous evidence linking dynamic balance in dancers to refined motor strategies and intersegmental control [25,26]. In competitive dance—where precision, single-leg support, and symmetry are crucial—such improvements have both performance and preventive relevance. The between-group differences confirm a protocol-specific effect on dynamic balance and limb symmetry.

#### 4.2.2. Pediatric Reach Test (Anticipatory Postural Control)

In the EG, PRT performance improved in all three directions (anterior and lateral; Figure 2a–c), suggesting enhanced anticipatory control and trunk–core stability. These adaptations are consistent with studies highlighting the efficacy of proprioceptive and core-based exercises in improving pre-activation timing and balance recovery in dancers and youth athletes [30,39,40,41,42]. The results point to a robust, intervention-specific effect on anticipatory postural adjustments.

#### 4.2.3. CMJ (Stretch–Shortening Explosive Strength)

The EG showed increases in right single-leg and bilateral CMJ height, with a non-significant trend for the left single-leg condition (Figure 3a–c). These results reflect the transfer of proprioceptive and plyometric stimuli to neuromuscular recruitment and kinetic-chain efficiency [35,43,44]. The observed changes represent small-to-moderate but functionally meaningful improvements in explosive lower-limb power, confirming the integrative potential of proprioceptive work in enhancing vertical force production.

#### 4.2.4. SLLST (Post-Landing Stabilization)

In the EG, stabilization times decreased bilaterally (Figure 4a,b), indicating improved impact absorption and faster balance recovery in dynamic single-leg conditions. This is crucial in dance, where repeated jumps, turns, and rapid support transitions require precise control of landing mechanics. Youth-athlete literature highlights the role of reduced post-landing instability as a marker of improved neuromuscular control [31,45]. Our approach using a lumbar accelerometer with a validated variance-threshold algorithm aligns with prior instrumental methods [13,32] and supports the training-specific improvement in postural re-stabilization capacity.

#### 4.2.5. Synthesis of ANOVA Findings and Test Sensitivity

Significant interactions for YBT, PRT, CMJ, and SLLST (Table 6) point to a coherent pattern of adaptations favoring the EG. Dynamic, multicomponent tests (YBT, SLLST, CMJ) appear more sensitive in detecting functional change in highly coordinated contexts such as dance than static measures—consistent with our aims and test battery.

### 4.3. Between-Group Comparison and Hypothesis Verification

Overall, the present findings align with previous evidence demonstrating that proprioceptive and neuromuscular programs enhance dynamic balance, symmetry, and landing control in youth and dancer populations [16,17,18,30,39]. The consistent improvements across tests suggest integrated sensorimotor adaptations, while the modest change in the left single-leg CMJ may reflect natural asymmetries and neuromuscular variability during adolescence. These adaptations are particularly relevant in this developmental stage, characterized by growth-related fluctuations in coordination and proprioceptive acuity, when structured balance training can support motor stability and injury prevention. Given the limited experimental literature on Latin dancesport, these results contribute novel evidence linking proprioceptive load progression with functional performance improvements in adolescent dancers.

### 4.4. Practical Implications

For dance teachers and strength and conditioning coaches, adding 25–30 min 2×/Week of progressive proprioceptive exercises (instability, controlled perturbations, reaching and multitasking) before the technical class can improve: (a) dynamic balance and symmetry (YBT), (b) anticipatory control (PRT), (c) jump power (CMJ), and (d) landing stabilization (SLLST). These findings support the systematic inclusion of structured balance workouts in dance schools, which often lack differentiated dynamic-balance training pathways.

### 4.5. Study Limitations

- Sampling and allocation. Convenience sampling from schools in Southern Italy and the absence of full randomization may introduce selection bias and unmodeled cluster effects (instructor/context differences).
- Examiner blinding. Likely lack of assessor blinding may have increased the risk of measurement bias.
- Instrumentation. Although objective measures were used (lumbar IMU for SLLST; Chronojump for CMJ), gold-standard systems (force platforms, 3D motion capture, EMG) were not employed. A single IMU cannot capture distal/proximal segmental contributions to postural control.
- Intervention duration and dose. The 10-week, 2×/week, 25–30 min cycle is pragmatic but does not establish dose–response relationships or longer-periodization effects.
- External validity. The sample comprised adolescent females practicing Latin American dance; generalizability to males, other dance styles, or age groups remains to be verified.
- Maturation and load. Maturational status (e.g., PHV/Tanner) and training load (e.g., weekly RPE) were not controlled, representing potential confounders in youth.
- Multiple comparisons and learning. Although primary outcomes were pre-specified and Holm–Bonferroni was applied, type I error cannot be fully excluded; residual learning effects cannot be ruled out (mitigated by the CG).
- Moreover, as all participants were recruited from Latin dance schools in a single geographic region of Southern Italy, the generalizability of the findings to other populations and contexts may be limited.

### 4.6. Future Directions

- Conduct randomized trials (preferably cluster-RCTs by school/instructor) with blinded assessors;
- Include medium/long-term follow-ups (≥3–6 months) to assess retention;
- Extend the population to males, other disciplines, and multiple centers, controlling for maturational status and limb dominance;
- Integrate advanced instrumentation (force platforms, 3D motion capture, surface EMG, multi-segment IMU networks) and dual-task/visuo-vestibular paradigms to stress anticipatory and reactive control;
- Test balance-training variants (perturbation-based, visuo-proprioceptive, progressive instability) and define dose–response and progressions;
- Monitor clinically relevant outcomes (injuries, training absences) and adherence/feasibility in school–sport settings.

## 5. Conclusions

The proprioceptive protocol integrated into dance practice produced specific and clinically meaningful improvements in dynamic balance (YBT), anticipatory postural control (PRT), post-landing stabilization (SLLST), and explosive strength (bilateral CMJ and right single-leg CMJ), together with reduced YBT functional asymmetry. Group × time interactions in the ANOVA [F(1,122), *η_P_*^2^ in the 0.04–0.26 range] indicate that the observed changes are attributable to the intervention rather than ordinary training.

Practically, integrating 25–30 min, twice weekly, of progressive proprioceptive work before the technical class is a low-cost, highly transferable strategy to enhance key performance components (symmetry, dynamic control, landing management) in adolescent dancers. These findings support the adoption of multimodal, periodized balance programs in school and club settings.

## Figures and Tables

**Table 1:** Baseline anthropometric characteristics at T0 (mean ± SD) by group: Experimental Group (EG) and Control Group (CG).

Characteristic	EG	CG
Age (years)	14.5 ± 0.6	14.7 ± 0.7
Stature (cm)	156.17 ± 8.09	156.50 ± 6.19
Body mass (kg)	50.50 ± 8.55	49.75 ± 7.48
BMI (kg/m^2^)	20.61 ± 2.67	20.23 ± 2.18

Note. Values are mean ± standard deviation (SD). Units: age in years; height in cm; body mass in kg; BMI in kg/m^2^. Abbreviations: BMI = body mass index; T0 = baseline (pre-test).

**Table 2:** Overall training structure and weekly progression across 10 weeks (Experimental Group, EG). The Control Group (CG) followed an unchanged technical/traditional program (see Note).

Week(s)	EG—Balance/Proprioceptive Add-on (25–30 min Before Class, 2×/Week)
1	Static and dynamic tasks on foam and air cushions; ball and elastic band drills; landing balance and the ‘airplane’ exercise (single-leg trunk/hip extension).
2	Progression of week 1 tasks; add rotations and eyes-closed variants.
3	Jumps on pads in line and zig-zag; balloon games; “airplane” on blocks; elastic-spring device; single-leg balance with weight transfers.
4	Progression of week 3 tasks (greater range, control, and hold time).
5	Return to cushion-based proprioception; ball exchanges; paired balance on blocks; squat jumps; lunge-to-jump transitions.
6	Progression of week 5 tasks; introduce competitive balance games and controlled destabilization.
7–8	Repeat weeks 1–2 protocol with higher postural precision and longer holds.
9	Repeat weeks 3–4 with added direction changes, unstable contacts, and reduced visual input.
10	Final combination: static, dynamic, cooperative, and eyes-closed sequences.

Note. CG (constant across weeks): technical/traditional training (2×/week, 90 min): muscular activation; revision of basic figures; study/refinement of combinations; choreographic sequences across the five dances; endurance simulation (competition-like); stretching and breathing. Training volume (matched): EG = 2×/week (25–30 min balance add-on) + 60 min standard class (~90 min/session; ~180 min/week); CG = 2×/week, ~90 min sessions (~180 min/week).

**Table 5:** Two-way repeated-measures ANOVA (group × time) for the interaction effect; interaction term reported as F(1,122). Effect size reported as partial eta-squared (*η_P_*^2^).

Variable	F(1,122)	*p*-Value	Sig.	*η* *_P_* ^2^
Composite Score Right (%)	43.534	<0.001	***	0.263
Composite Score Left (%)	25.929	<0.001	***	0.175
PRT_Anterior (cm)	19.284	<0.001	***	0.136
PRT_Lateral Right (cm)	16.357	<0.001	***	0.118
PRT_Lateral Left (cm)	10.327	0.004	**	0.078
CMJ_Single Right (cm)	7.676	0.011	*	0.059
SLLST_Right (s)	6.190	0.021	*	0.048
SLLST_Left (s)	5.315	0.031	*	0.042
CMJ (cm)	5.307	0.031	*	0.042
Asymmetry Composite (%)	4.858	0.038	*	0.038

Note. Two-way repeated-measures ANOVA (group × time); interaction term reported as F(1,122) with *η_P_*^2^ (partial eta-squared) as effect size. Units: as in Table 4. Abbreviations: YBT = Y Balance Test; PRT = Pediatric Reach Test; CMJ = Countermovement Jump; SLLST = Single-Leg Landing Stability Test; EG = Experimental Group; CG = Control Group. Variables Composite Score Right, Composite Score Left, and Asymmetry Composite refer to YBT-derived measures of dynamic balance. Significance levels: * *p* < 0.05; ** *p* < 0.01; *** *p* < 0.001.

**Table 6:** Paired comparisons (T0 vs. T1) within groups (EG, CG) using paired *t*-tests; effect sizes reported as Cohen’s d_z.

Variable	Group	*t* (T0 vs. T1)	*p*-Value	Sig.	Cohen’s d_z
Composite Score Right (%)	EG	−10.214	<0.001	***	−1.297
Composite Score Right (%)	CG	−0.135	0.895	n.s.	−0.017
Composite Score Left (%)	EG	−6.847	<0.001	***	−0.870
Composite Score Left (%)	CG	−1.191	0.259	n.s.	−0.151
Asymmetry Composite (%)	EG	−2.350	0.038	*	−0.298
Asymmetry Composite (%)	CG	1.014	0.332	n.s.	0.129
PRT_Anterior (cm)	EG	−4.088	0.002	**	−0.519
PRT_Anterior (cm)	CG	1.609	0.136	n.s.	0.204
PRT_Lateral Right (cm)	EG	−3.391	0.006	**	−0.431
PRT_Lateral Right (cm)	CG	2.379	0.037	*	0.302
PRT_Lateral Left (cm)	EG	−3.179	0.009	**	−0.404
PRT_Lateral Left (cm)	CG	0.895	0.390	n.s.	0.114
CMJ_Single Right (cm)	EG	−4.883	<0.001	***	−0.620
CMJ_Single Right (cm)	CG	0.229	0.823	n.s.	0.029
CMJ_Single Left (cm)	EG	−1.194	0.258	n.s.	−0.152
CMJ_Single Left (cm)	CG	1.276	0.228	n.s.	0.162
CMJ (cm)	EG	−2.327	0.040	*	−0.296
CMJ (cm)	CG	0.678	0.512	n.s.	0.086
SLLST_Right (s)	EG	3.711	0.003	**	0.471
SLLST_Right (s)	CG	−0.172	0.866	n.s.	−0.022
SLLST_Left (s)	EG	3.246	0.008	**	0.412
SLLST_Left (s)	CG	−0.538	0.601	n.s.	−0.068

Note. Paired comparisons (Δ = T0–T1) using paired *t*-tests; Cohen’s d_z_ = t/√n (n = 62). Units: as in Table 4. Variables Composite Score Right (%), Composite Score Left (%), and Asymmetry Composite (%) refer to YBT-derived measures of dynamic balance. For SLLST, lower times indicate better performance; with Δ = T0–T1, improvements on YBT/PRT/CMJ yield negative t and d_z_, whereas improvements on SLLST yield positive values. Significance: n.s. = not significant; * *p* < 0.05; ** *p* < 0.01; *** *p* < 0.001. Abbreviations: YBT = Y Balance Test; PRT = Pediatric Reach Test; CMJ = Countermovement Jump; SLLST = Single-Leg Landing Stability Test; EG = Experimental Group; CG = Control Group.

**Table 7:** Key statistical results: within-group (paired *t*-test, EG) and group × time interaction (two-way repeated-measures ANOVA).

Variable	Test Type	Statistic	*p*-Value	Effect Size	Sig.	Interpretation
Composite Score Right (YBT)	Paired *t*-test (EG)	*t* = −10.214	<0.001	d_z = −1.297	***	Very large improvement
Composite Score Left (YBT)	Paired *t*-test (EG)	*t* = −6.847	<0.001	d_z = −0.870	***	Large improvement
Asymmetry Composite (YBT)	Paired *t*-test (EG)	*t* = −2.350	0.038	d_z = −0.298	*	Significant asymmetry reduction
PRT—Anterior (cm)	Paired *t*-test (EG)	*t* = −4.088	0.002	d_z = −0.519	**	Significant improvement
PRT—Lateral Right (cm)	Paired *t*-test (EG)	*t* = −3.391	0.006	d_z = −0.431	**	Significant improvement
PRT—Lateral Left (cm)	Paired *t*-test (EG)	*t* = −3.179	0.009	d_z = −0.404	**	Significant improvement
CMJ_Single Right (cm)	Paired *t*-test (EG)	*t* = −4.883	<0.001	d_z = −0.620	***	Significant jump-height increase
CMJ (cm)	Paired *t*-test (EG)	*t* = −2.327	0.040	d_z = –0.296	*	Significant improvement
SLLST Right (s)	Paired *t*-test (EG)	*t* = 3.711	0.003	d_z = 0.471	**	Shorter stabilization time (better)
SLLST Left (s)	Paired *t*-test (EG)	*t* = 3.246	0.008	d_z = 0.412	**	Shorter stabilization time (better)
Composite Score Right (YBT)	ANOVA (group × time)	F(1,122) = 43.534	<0.001	*η_P_*^2^ = 0.263	***	Significant interaction
Composite Score Left (YBT)	ANOVA (group × time)	F(1,122) = 25.929	<0.001	*η_P_*^2^ = 0.175	***	Significant interaction
Asymmetry Composite (YBT)	ANOVA (group × time)	F(1,122) = 4.858	0.038	*η_P_*^2^ = 0.038	*	Significant interaction
PRT—Anterior (cm)	ANOVA (group × time)	F(1,122) = 19.284	<0.001	*η_P_*^2^ = 0.136	***	Significant interaction
PRT—Lateral Right (cm)	ANOVA (group × time)	F(1,122) = 16.357	<0.001	*η_P_*^2^ = 0.118	***	Significant interaction
PRT—Lateral Left (cm)	ANOVA (group × time)	F(1,122) = 10.327	0.004	*η_P_*^2^ = 0.078	**	Significant interaction
CMJ_Single Right (cm)	ANOVA (group × time)	F(1,122) = 7.676	0.011	*η_P_*^2^ = 0.059	*	Significant interaction
CMJ (cm)	ANOVA (group × time)	F(1,122) = 5.307	0.031	*η_P_*^2^ = 0.042	*	Significant interaction
SLLST Right (s)	ANOVA (group × time)	F(1,122) = 6.190	0.021	*η_P_*^2^ = 0.048	*	Significant interaction
SLLST Left (s)	ANOVA (group × time)	F(1,122) = 5.315	0.031	*η_P_*^2^ = 0.042	*	Significant interaction

Note. Paired comparisons report Cohen’s d_z_ = t/√n (n = 62). ANOVA interaction effects report η_P_^2^. Units: as in Table 4. For SLLST, lower times indicate better performance; the sign of t and d_z_ follows the Δ = T0–T1 convention. Abbreviations: YBT = Y Balance Test; PRT = Pediatric Reach Test; CMJ = Countermovement Jump; SLLST = Single-Leg Landing Stability Test; EG = Experimental Group. Sig.: * *p* < 0.05; ** *p* < 0.01; *** *p* < 0.001.

## Data Availability

The data presented in this study are available on request from the corresponding author.

## Abbreviations

The following abbreviations are used in this manuscript:

EG	Experimental Group
CG	Control Group
YBT	Y Balance Test
PRT	Pediatric Reach Test
SLLST	Single-Leg Landing Stability Test
CMJ	Countermovement Jump
T0	Baseline (pre-test)
T1	Post-test
LL	Lower Limb Length
ASIS	Anterior Superior Iliac Spine
IMU	Inertial Measurement Unit
ICC	Intraclass Correlation Coefficient
η*_P_*^2^	Partial Eta Squared
SOP	Standard Operating Procedure
